# Twenty-fifth Annual Report of the Bloomingdale Asylum for the Insane

**Published:** 1846-03

**Authors:** 


Art. V.—*Twenty-Fifth Annual Report of the Bloomingdale Asylum for the Insane.* For the year 1845. By Pliny Earle, M. D., Physician to the Asylum.

*Report of the Pennsylvania Hospital for the Insane.* For the year 1845. By Thomas S. Kirkbride, M. D., Physician to the Institution.

In these reports we find the usual amount of statistics, showing that good men are still successfully laboring to ameliorate the condition of the insane. Dr. Earle states that at the Bloomingdale Asylum—

"The year was commenced with one hundred and four patients, of whom fifty-four were males and fifty females. Since that time, one hundred and thirty-eight cases, of which seventy-one were males and sixty-seven females, have been admitted, making the whole number of cases under treatment during the year two hundred and forty-two, of which one hundred and twenty-five were males and one hundred and seventeen females.

"One hundred and thirteen cases,—fifty-eight males and fifty-five females,—have been discharged. Seven males and five females have died, leaving now in the Asylum one hundred and seventeen patients, of whom sixty are males and fifty-seven females.

"Of the cases discharged, sixty-one were cured, twelve much improved, twenty improved and twenty unimproved, being discharged at the request of their friends.

"Four of the patients who are recorded as much improved, became entirely well soon after their removal. Several others who were believed to be curable, were prematurely taken away, thus losing the advantage which might have accrued from a longer residence in the Asylum."

Among other substantial reasons why more ample provision for the insane should be made, in every part of the country, is the unquestionable fact that madness is becoming a more frequent malady among our people. The following are Dr. Earle's remarks on this subject:

"The number of patients admitted during the past year, as compared with the annual admissions for several of the preceding years, being assumed as the data upon which to found an opinion, the necessary inference is, that mental disorders are increasing. Whether the increase be in a greater ratio than that of the population of the City and its adjacent country, is a proposition which cannot easily be demonstrated. However this may be, it is an unquestionable fact, that the exciting causes of mental alienation were never, in time of peace, more active, among any people, than at the present day among the inhabitants of the United States; and particularly so in the States which, bordering on the Atlantic, were the earliest peopled by European emigrants.

"Intoxicating liquors are so cheap that the labor of a few hours will procure enough to addle the brain for a week, and prevent the healthy exercise of reason perhaps a much longer period. The avenues to wealth, place and power, are open to all: the child of the cottager thus entering into the strife of competition with the son of the most wealthy citizen. The progress of civilization and refinement, and the comparative ease with which the products of both nature and art in every quarter of the globe are here obtained, have a direct tendency to foster a luxurious life. Hence human desires and human wants are greatly multiplied, while both mind and body are exerted to the utmost power of endurance to gratify the former and supply the latter. The almost unavoidable effect of the artificial mode of living thus produced, is either a debility of the system, or an augmentation of nervous excitability, either of which facilitates the invasion of mental disease.

Art has made advances so rapid towards the annihilation of time and space, that, if life be measured by the proper standard—the number of events, circumstances and conditions, seen, felt or perceived—the amount of pleasure enjoyed and of pain endured—the people of the present generation have an existence of ten-fold longer duration than their forefathers. As if this were not enough, the mind is forced into an activity corresponding with the new era of art.—Children, before the body has acquired sufficient tone, or the brain sufficient firmness, to endure much mental exertion with impunity, are placed in schools where the intellectual faculties are unduly urged, while the physical exercise necessary to the due development of the frame is too often neglected. Under such circumstances, the head will expand, but the body cannot grow in size or vigor sufficiently to maintain 'a balance of power.'"

The age at which derangement comes on is found to be earlier in New York than in Europe. The statement of Dr. Earle on that head is given in the subjoined extract:

"Some European authors maintain that insanity prevails to a greater extent in persons between the ages of 30 and 40 years than in any other decennial period of life. It is proved, however, that in this country the first invasion of the disease is the most frequent in the decade between 20 and 30 years. This fact is corroborated by the cases in the foregoing table. Forty-two of the patients were, at the time of admission, between 21 and 30 years, inclusive, and but twenty-eight, from 31 to 40. At the time of first attack, forty-four were from 21 to 30, and but twenty-seven from 31 to 40."

The relative frequency in the two sexes is thus stated:

"Of males, the single exceed the married, exclusive of widowers, by one. On the contrary, of females, the married exceed the single by eight. Of both sexes, the number of the married is seven greater than that of the single. This does not accord with the general rule deduced from a large number of cases. Of 5,322 patients, of both sexes, received into twelve institutions, 2,777 were single, 2,120 married, and 425 (135 men and 290 women) widowed. Here the number of the single exceeds that of the married, by 657, or very nearly 31 per cent. It is generally admitted that among the insane there are more unmarried than married, and it is a current opinion that prolonged celibacy increases the tendency to mental derangement.

"The number of widowed females insane, is generally much larger than that of widowed males. It will be perceived that twice as many of the former as of the latter were received during the year, and that in the statistics from twelve asylums, the former exceed the latter more than 100 per cent."

Dr. Earle's experience is favorable to the curableness of insanity in persons advanced in years. He says:

"It is generally believed that insanity is curable in a proportion inverse to that of the ages of the patients. This may, to a certain extent, be correct, but it is probable that the rule is of far less general application than is usually supposed. By consulting the table, it will be perceived that several of the persons who recovered, were somewhat advanced in life.'

The proportion of cures effected is reported in the annexed paragraph:

In Mania 50 per cent. were cured, in Melancholia 57 per cent., in partial Mania 25 per cent., and in Dementia 20 per cent. The number of the patients, however, is so small that it would be unsafe to make general rules from these results. Insanity is not generally susceptible of a rapid cure. The shock to the system is so great that, frequently, even if the patient be brought to the Asylum soon after the commencement of the disease, several months are requisite to restore it to its natural condition.

"There is a strong tendency in the disease, from the time of its invasion, to assume the chronic form; and if this be assumed, the case becomes more difficult of cure. The practical lesson to be learned from this fact, is the high importance of placing the patient under a judicious course of curative treatment at as early a period as possible."

To this, the three facts which Dr. Earle urges, with emphasis, ought to be added, and impressed by the profession upon the community: namely—

"1st. As a general rule, the first measure in the curative treatment of insanity, is to remove the patient from home, from acquaintances, and from all familiar scenes and associations.

"2nd. When the insane are placed under proper curative treatment in the early stages of the disease, from 75 to 90 per cent. recover.

"3rd. On the contrary, if they be not put under treatment before the disease has continued a year or more, from 15 to 20 cent. only, are cured."

We learn from Dr. Kirkbride's report, that the average number of patients in the Pennsylvania Hospital during the past year was 162, and that of these 159 were discharged—80, or one half, cured, 5 much improved, 24 improved, and only 30 stationary, 20 having died. Of the patients discharged cured, 39 were residents of the hospital not exceeding three months; 26 between three and six months; 12 between six months and a year, and 3 for a longer period than a year.

"Of those discharged 'much improved,' one was under treatment less than three months, three between three and six months, and one for more than a year.

"Of the 'improved,' six were under care less than six months, three between three and six months, ten between six months and one year, and five for more than one year.

"Of those discharged and reported 'stationary,' eight were under care less than three months, seven between three and six months, six between six months and one year, and nine for a longer period than one year."

"Make a melancholy man fat," says old Burton, "and you effect a cure." It was found so at the Pennsylvania Hospital. Dr. K. says:

"Among the *discharges*, have been numerous cases of great interest, many whose recoveries have been very prompt, and several who have regained their health under the most discouraging circumstances. The change in appearance and great increase of weight in some of these, have been such as often to attract attention from all whose official duties brought them in contact with the patients. It has been quite common for an individual to gain twenty or thirty pounds during the period of convalescence and one patient who became perfectly well, increased sixty pounds in his weight in less than two months, a part of the time having an average gain of more than two pounds a day. When most emaciated,—weighing only eighty-seven pounds at one time,—he had a voracious appetite, ate very freely, but lost flesh constantly, and yet manifested no symptom of disease, except high mental excitement. As his insanity subsided, the nutritive functions resumed their healthful condition, and he weighed one hundred and forty-seven before he left us. In a few weeks after his discharge he paid the hospital a visit and had then reached one hundred and sixty pounds, and his acquaintances of a few months before, were quite excusable for not recognising in his portly form, the skeleton-like individual who had been their associate."

The following picture from Dr. K.'s pen shows how many are the comforts that may be thrown around the prison-house of even the hopelessly insane:

"Of the patients who *remain*, are a large number of curable cases and many whose stay in the Institution will be short. Near one hundred, however, are individuals who must, in all human probability, look to this or some similar establishment as their permanent home, and the spot in gathering around which every source of comfort and happiness, they have a deeper interest than in any other. Many of them have already been residents of this hospital and of that in the City of Philadelphia, ten, twenty or thirty years; one gentleman has been under the care of the Institution more than forty-one years, and a lady more than fifty-five years. Both these last are still blessed with an abundant share of physical health, and scarce look older than they did when I first knew them, fifteen years ago. The gentleman retains all the courtesy of character, polished manner and social disposition which eminently characterised him in youth, and which still make him one of the most welcome guests at all our parties and entertainments. In the lady, although her intellect may have suffered more, the affections still flourish in all their original vigor, and she is noted for her kindness, her warmth of feeling, and tender sympathy for all who suffer, and she has Christian sources of consolation, sufficient to atone for all her afflictions. Two others have been forty-two and forty-five years in the hospital.

